# M. Pigeau’s Case of Asphyxia Dissipated by a Pinch of Snuff

**Published:** 1842-05-07

**Authors:** 


*Asphyxia dissipated by a pinch of Snuff.*—A child, five months old, being constipated, was given a spoonful of sweet oil. The dose having been carelessly administered, some of the oil got down the teachea, and brought on complete asphyxia; the lungs had ceased to contract, and the infant was on the point of death. M. Pigeau, who was sent for, at once perceived the cause of this alarming state, and passed a pinch of snuff up the nostrils of the infant. The violent sneezing thus brought on excited the contractibility of the respiratory muscles, and respiration commenced, feebly at first, but was gradually re-established. Strong vinegar and other means had been previously tried, but without without success.—*Provincial Med. and Surg. Journal.* March 12th, 1842.

